# Mesh quality comparison of common three file formats derived from intraoral scans

**DOI:** 10.1007/s00784-026-06946-0

**Published:** 2026-05-29

**Authors:** Mahmoud E. Elbashti, Gülce Çakmak, Marta Macarena Paz-Cortes, Nadin Al-Haj Husain, Samir Abou-Ayash, Andrea Roccuzzo, Pedro Molinero-Mourelle

**Affiliations:** 1 Department of Advanced Prosthodontics, Graduate School of Medical and Dental Sciences, Institute of Science Tokyo, 1-5-45, Yushima, Bunkyo-ku, Tokyo 113-8549 Japan; 2 Department of Reconstructive Dentistry and Gerodontology, School of Dental Medicine, University of Bern, Bern, Switzerland; 3 Department of Prosthodontics, Geriatric Dentistry and Craniomandibular Disorders, Corporate Member of Freie Universität Berlin and Humboldt-Universität zu Berlin, Charité- Universitätsmedizin Berlin, Berlin, Germany; 4 Department of Dental Clinical Specialties, Faculty of Dentistry, Complutense University of Madrid, Madrid, Spain; 5 Unit for Practice-based Research, School of Dental Medicine, University of Bern, Bern, Switzerland; 6 Department of Prosthetic Dentistry, University Medical Center of the Johannes Gutenberg- University Mainz, Mainz, Germany; 7 Shanghai Perio-Implant Innovation Center, Institute of Integrated Oral, Craniofacial and Sensory Research, Shanghai Ninth People’s Hospital, Shanghai Jiao Tong University School of Medicine, 4F Building 1, 115 Jinzun Road, Pudong Research Campus, Shanghai, China; 8 College of Stomatology, Shanghai Jiao Tong University, National Center of Stomatology, National Clinical Research Center for Oral Diseases, Shanghai Key Laboratory of Stomatology, Shanghai, China; 9 Department of Periodontology, School of Dental Medicine, University of Bern, Bern, Switzerland; 10 Department of Conservative Dentistry and Prosthodontics, Faculty of Dentistry, Complutense University of Madrid, Madrid, Spain; 11 Complutense University of Madrid, Ramon y Cajal Research Institute (IRYCIS), Madrid, Spain; 12 Restorative Dental Sciences, Faculty of Dentistry, the University of Hong Kong, Hong Kong SAR, China

**Keywords:** Intraoral scanning, Mesh quality, File format conversion, Digital dentistry, Geometric accuracy

## Abstract

**Purpose:**

To comparatively evaluate mesh quality metrics in PLY, STL, and OBJ files derived from native intraoral scans and exported using different mesh processing software, with emphasis on computational performance, geometric fidelity, and mesh resolution.

**Methods:**

Fifty maxillary and mandibular full-arch intraoral scan datasets were acquired using a Trios 4 intraoral scanner under clinical conditions. Native datasets were exported directly in PLY format without decimation, smoothing, or mesh optimization and designated as reference files. Each reference dataset was converted into PLY, STL, and OBJ formats using four mesh processing software packages (Meshmixer, Blender, MeshLab, and CloudCompare) under default export settings without mesh-altering procedures. Quantitative mesh evaluation was performed in MeshLab, including computational performance (loading time, export time, and file size), geometric accuracy (bounding box dimensions and surface area), and mesh resolution (total edge count and average edge length). Statistical analyses were conducted to compare differences among file formats and software platforms.

**Results:**

Computational performance differed significantly across file formats (*p* < 0.001). Native PLY files demonstrated the fastest loading (median 293 MS) and export times (median 627 MS), whereas OBJ files exhibited the longest export durations (median 51,639 MS) and largest file sizes. STL files showed intermediate performance. In contrast, bounding box dimensions and surface area measurements showed no significant differences between reference and converted files (*p* = 1.000), indicating preservation of scale and anatomical coverage. Edge-based metrics also revealed no significant differences in total edge count or average edge length (*p* = 1.000), confirming maintenance of mesh resolution and tessellation density.

**Conclusion:**

File format significantly influences computational efficiency but does not affect geometric accuracy or mesh resolution when default export parameters are applied. PLY format demonstrated superior workflow performance while maintaining equivalent geometric and topological integrity across software platforms.

**Clinical significance:**

Under the specific conditions of this study, PLY, STL, and OBJ formats preserved global geometric properties during conversion, while PLY demonstrated superior computational efficiency and smaller file size. Within these constraints, the PLY format may be considered a preferred export option for intraoral scan data, as it can help streamline digital workflows in prosthodontics, orthodontics, and restorative dentistry, reducing technical bottlenecks and improving efficiency. However, clinicians and dental technicians may still exchange PLY, STL, and OBJ formats without concern for scaling distortion, and caution is warranted when generalizing these findings to other systems, non-default settings, or workflows involving mesh modification.

## Introduction

The digital dentistry workflow continues to expand, driven by the rapid development and continuous refinement of advanced digital technologies [1]. These include intraoral scanners, cone-beam computed tomography (CBCT), facial scanning systems, computer-aided design and computer-aided manufacturing (CAD/CAM) technologies, as well as functional and aesthetic evaluation tools. Collectively, these innovations have redefined diagnostic and therapeutic approaches, facilitating comprehensive digital integration across clinical and laboratory environments [2–5].

Among these technologies, intraoral scanners have become an integral component of daily clinical practice. They enable high-resolution three-dimensional (3D) acquisition of dental and oral structures with enhanced efficiency, reproducibility, and patient comfort compared with conventional impression techniques [6]. The resulting digital datasets serve as the foundation for CAD/CAM workflows, virtual treatment planning, additive manufacturing, and interdisciplinary data exchange. Therefore, the structural integrity, geometric fidelity, and computational performance of exported mesh files are critical determinants of accuracy, workflow efficiency, and overall clinical predictability within contemporary digital dental practice.

**Fig. 1:**
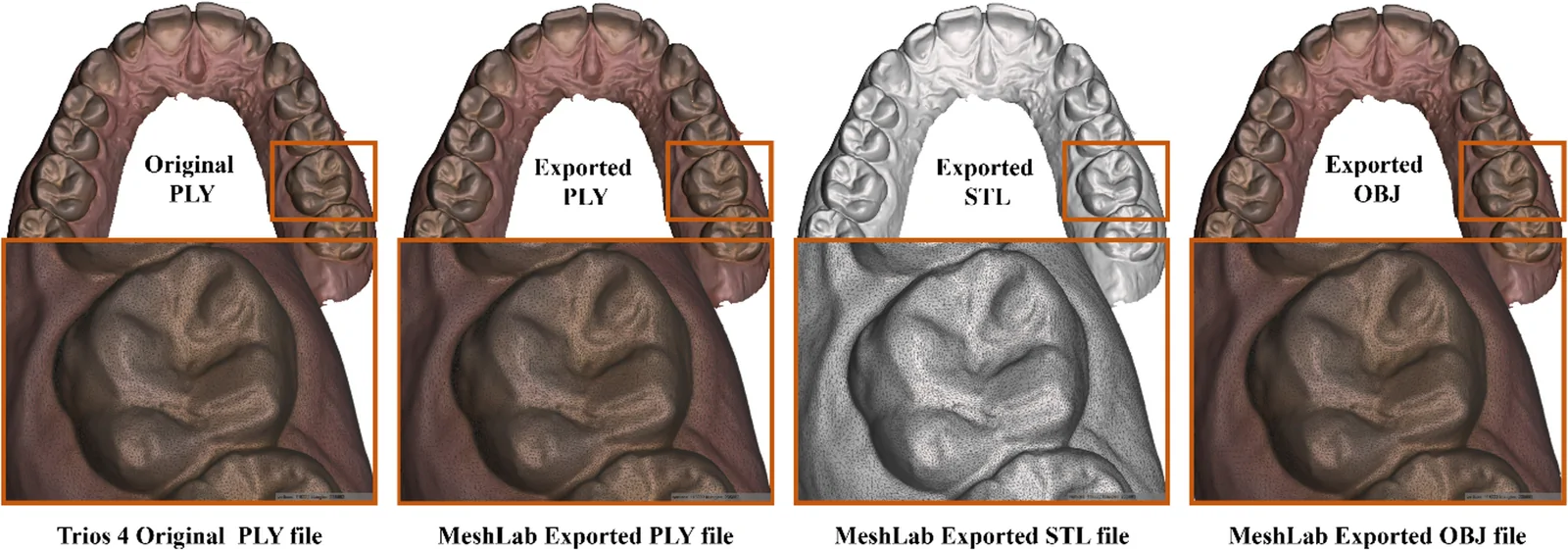
Representative comparison of a maxillary full-arch intraoral scan in original native PLY format and after export to PLY, STL, and OBJ using MeshLab. Upper row: full-arch views; lower row: magnified occlusal region (orange boxes). No visible differences in surface morphology or detail are observed among formats, indicating preservation of geometric fidelity after conversion

Intraoral scanners generate surface tessellations that represent dental anatomy as polygonal meshes composed of vertices, edges, and faces. In principle, when the same mesh topology is preserved, geometric measurements derived from these representations should be consistent regardless of file format. These meshes are commonly exported in standard formats such as polygon file format (PLY), stereolithography (STL), and object file (OBJ), which differ in geometric representation, encoding strategy, and metadata support (Fig. 1) [7]. STL encodes surfaces exclusively as collections of triangular facets and is typically implemented in either ASCII or binary form, prioritizing simplicity and compatibility with additive manufacturing and computer-aided design workflows. However, it is inherently limited to geometry-only data and does not store color or other attributes [8]. In contrast, PLY and OBJ support more flexible polygonal representations and can encode additional information such as vertex color, texture coordinates, and surface normals, with options for both ASCII and binary storage (PLY) or ASCII-based definitions with linked material files (OBJ). These features make PLY and OBJ more suitable for visualization-oriented applications, including shade analysis, soft tissue assessment, and comprehensive diagnostic documentation. Importantly, all three formats can encode the same vertex coordinates and face connectivity. Therefore, when mesh-altering operations are not applied, file format conversion is theoretically expected to preserve the underlying geometric shape. Although these formats are often treated as interchangeable within digital dental workflows, their differing roles as well as variations in encoding precision and software-specific export algorithms, may influence computational performance, storage efficiency, and mesh integrity [9].

In addition to proprietary CAD/CAM environments, free and open-source mesh modeling and processing software have become increasingly integrated into digital dental workflows [10, 11]. Platforms such as Meshmixer [12–15], Blender [16, 17], MeshLab [18, 19], and CloudCompare [15, 18, 20] are widely used for visualization, mesh inspection, segmentation, repair, format conversion, and accuracy evaluation. Their accessibility and flexibility make them valuable tools in both academic research and clinical practice, particularly for data preprocessing, accuracy evaluation, and interdisciplinary communication. However, variations in internal encoding strategies, default export parameters, and file writing algorithms across these platforms may introduce subtle but clinically relevant differences in mesh characteristics, even when no explicit mesh-altering operations are applied.

Despite the widespread clinical use of these file formats and software platforms, limited evidence exists regarding whether file conversion and export through different mesh processing software alter geometric fidelity or mesh resolution. Previous studies have primarily focused on scanner accuracy rather than post-acquisition file handling and format-dependent variability [21–25]. Moreover, variations in export implementation across commonly used software platforms may introduce subtle changes in mesh structure, potentially affecting downstream applications such as computer-aided design or 3D printing. In the local clinical and academic digital workflow, PLY, STL, and OBJ files are routinely exchanged between intraoral scanners, open-source mesh editors, and laboratory software without standardized validation of mesh preservation following conversion.

Evidence suggests that differences in file structure and encoding can affect computational efficiency in 3D data processing environments [9]. Therefore, the objective of this study was to comparatively evaluate computational performance, geometric accuracy, and mesh resolution of PLY, STL, and OBJ files derived from native intraoral scans and exported using different free and open-source mesh processing software. The aim was to determine whether file format and exporting software influence mesh quality metrics relevant to digital dental workflows. The null hypothesis was that neither file format nor exporting software would produce statistically significant differences in computational performance, geometric fidelity, or mesh resolution.

## Materials and methods

**Fig. 2:**
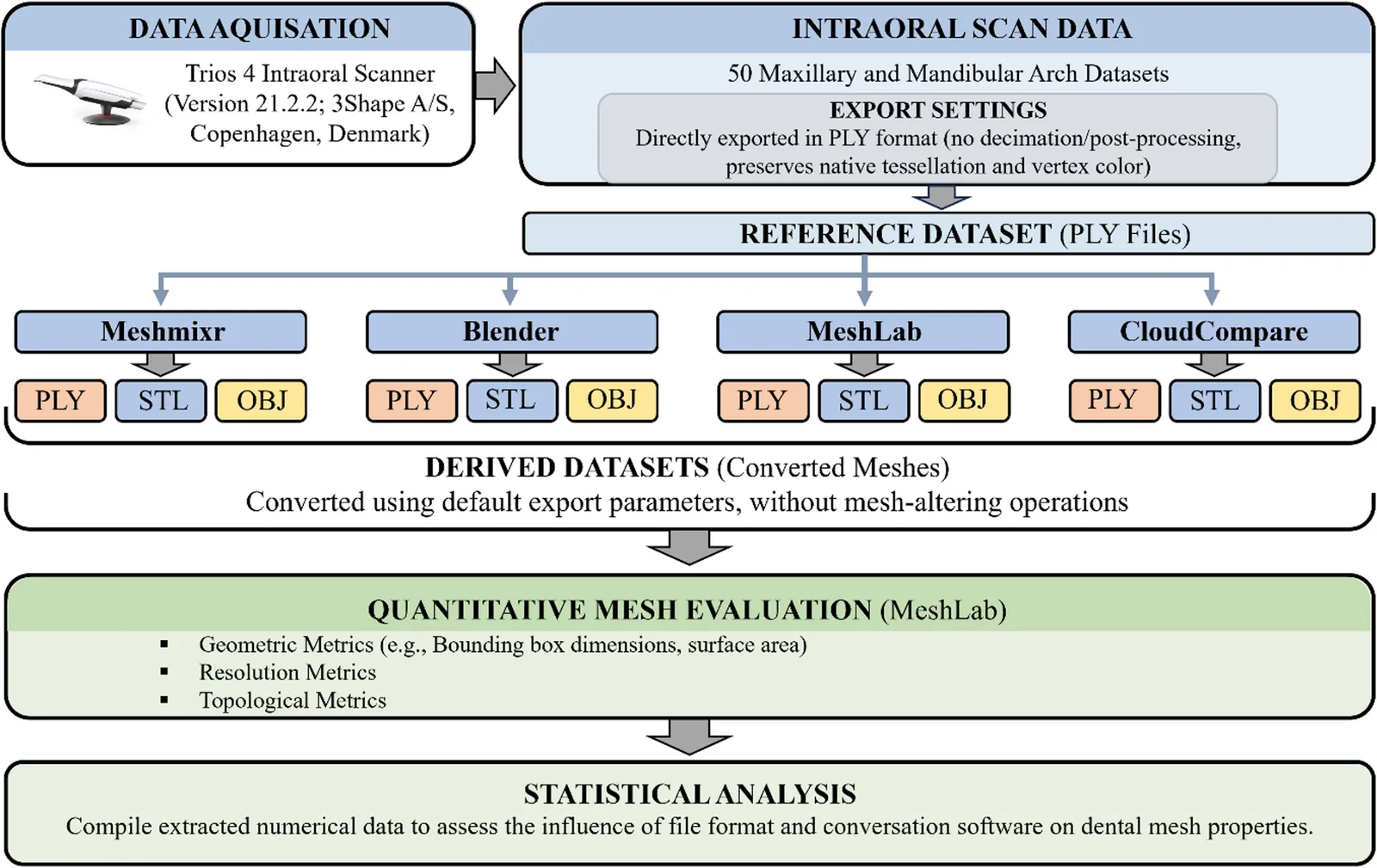
A flowchart summarizing the data acquisition, processing, and mesh metrics criteria evaluation for exported PLY, STL, and OBJ file formats using four different conversion and mesh processing software

A flowchart summarizing the data acquisition, processing, and mesh metrics criteria evaluation for exported PLY, STL, and OBJ file formats using four different conversion and mesh processing software is shown in Fig. 2.

### Data acquisition and processing

A total of 50 maxillary and mandibular intraoral scan datasets were included in this study. All scans were acquired using an intraoral scanner (Trios 4, software version 21.2.2; 3Shape A/S, Copenhagen, Denmark). The scanned data captured full-arch with associated color information. Each dataset was exported directly from the scanner software in PLY format without applying any decimation, smoothing, or mesh optimization procedures. The original PLY files, which preserved the native tessellation and vertex color data produced by the scanner, were designated as the reference datasets for subsequent analyses. The original reference PLY datasets were converted into PLY, STL, and OBJ formats using four different mesh processing software packages: Meshmixer (Autodesk Inc., USA), Blender (Blender Foundation, The Netherlands), MeshLab (ISTI–CNR, Italy), and CloudCompare (EDF R&D, France) (Table 1). All file conversions were performed using default export parameters, with explicit confirmation that no mesh decimation, remeshing, smoothing, or topology repair operations were applied. The converted meshes were treated as derived datasets and compared against the original PLY reference files to assess the influence of file format and conversion software on mesh characteristics.

**Table 1:** Comparison of software used for file conversion and mesh processing

Software	Version / Developer	Supported Export Formats	Primary role in study	Dental-Relevant Notes
Meshmixer	Version: 3.5.474 / Autodesk Inc., USA	PLY, STL, OBJ	File conversion	Popular in dental workflows but may apply hidden mesh optimizations if not carefully controlled.
Blender	Version: 4.5.3 LTS / Blender Foundation, Amsterdam, The Netherlands	PLY, STL, OBJ	File conversion	General-purpose 3D software; requires careful control to avoid unintended mesh processing.
MeshLab	Version: 2025.07 / ISTI–CNR, Pisa, Italy	PLY, STL, OBJ	File conversion and primary mesh analysis	Widely used in academic mesh analysis; provides standardized geometric and triangle quality metrics.
CloudCompare	Version: 2.14 alpha / EDF R&D, France	PLY, STL, OBJ	File conversion	Commonly used for dental accuracy and surface deviation studies; preserves scale and precision.

### Mesh metrics and evaluation criteria

To ensure terminological clarity, key concepts related to geometric evaluation were explicitly defined in this study. “Scale consistency” refers to the preservation of overall object dimensions, as assessed by bounding box measurements. “Geometric fidelity” denotes the maintenance of global shape characteristics, evaluated through metrics such as surface area. “Geometric accuracy,” in the context of this study, describes the degree of agreement between converted meshes and the reference dataset, rather than absolute correspondence to true anatomical geometry. For practical interpretation, preservation of shape was defined based on the stability of multiple quantitative descriptors, including unchanged bounding box dimensions, consistent surface area, and similar total edge count and average edge length across file formats. Together, these criteria provide a standardized framework for assessing whether file format conversion introduces measurable geometric alterations.

Quantitative mesh evaluation was performed to assess computational performance, geometric fidelity, mesh resolution, and topological integrity across file formats and conversion software. Only mesh properties that are characteristically comparable between PLY, STL, and OBJ formats and independent of format-specific metadata were included. All analyses were conducted using MeshLab and no mesh-altering operations were applied prior to measurement. Computational performance metrics were evaluated. File loading and opening time for each mesh were also recorded during import into MeshLab to assess differences in file parsing efficiency between PLY, STL, and OBJ formats. In addition, file export time required to convert the original reference PLY datasets into PLY, STL, and OBJ formats was measured for each software platform. Loading and export times were recorded in milliseconds (MS) and compared across file formats and software tools as indicators of workflow efficiency and practical usability in digital dental applications. Furthermore, the file size was recorded in kilobytes, (KB) at each stage of the workflow and compared across file formats and software tools as indicators of storage efficiency and practical usability in digital dentistry workflow.

Geometric accuracy and scale consistency were evaluated using bounding box dimensions along the X, Y, and Z axes. These metrics were used to verify that file conversion did not introduce scaling distortions. Surface area (mm²) was also computed as a global descriptor of anatomical coverage and geometric preservation, particularly relevant for full-arch dental scans. In addition, mesh resolution and tessellation density were assessed using edge-based metrics, including total edge count and average edge length. Average edge length was interpreted as an indirect indicator of local sampling density and the ability of the mesh to represent fine dental anatomical features such as cusps, fissures, and preparation margins. As intraoral scans represent open surface shells, meshes were expected to be non-watertight, and volumetric measurements were therefore excluded.

### Statistical analysis

All statistical analyses were performed using IBM SPSS Statistics for Windows (Version 25.0.0.0; IBM Corp., Armonk, NY, USA). Descriptive statistics, including measures of central tendency and dispersion, were calculated for all mesh-related variables. Given the distribution characteristics of the data, non-parametric tests were applied for analysis. The Kruskal–Wallis test was used for multiple group comparisons, while pairwise comparisons between the original reference PLY datasets and the derived PLY, STL, and OBJ files generated by each software platform were conducted using the Mann–Whitney U test. To control the potential type I error inflation associated with multiple intra-group and inter-group comparisons, Bonferroni correction was applied where appropriate. The level of statistical significance was set at 5% (α = 0.05) for all analyses.

## Results

### Computational performance metrics

**Fig. 3:**
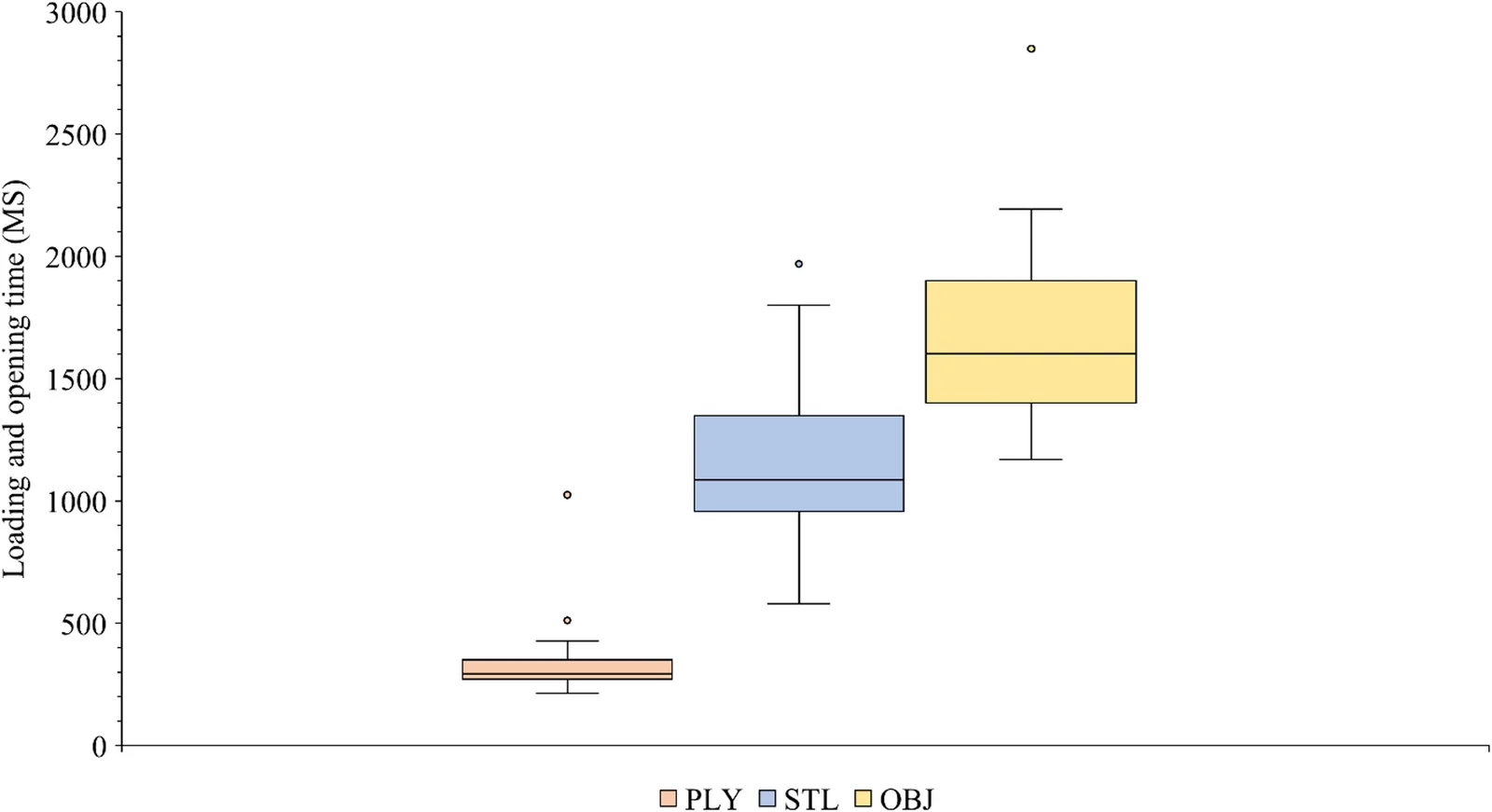
Boxplots of loading times (MS) for PLY, STL, and OBJ formats. Boxes show interquartile ranges with medians; whiskers indicate range and dots represent outliers. PLY loaded fastest, whereas OBJ showed the longest loading times

**Fig. 4:**
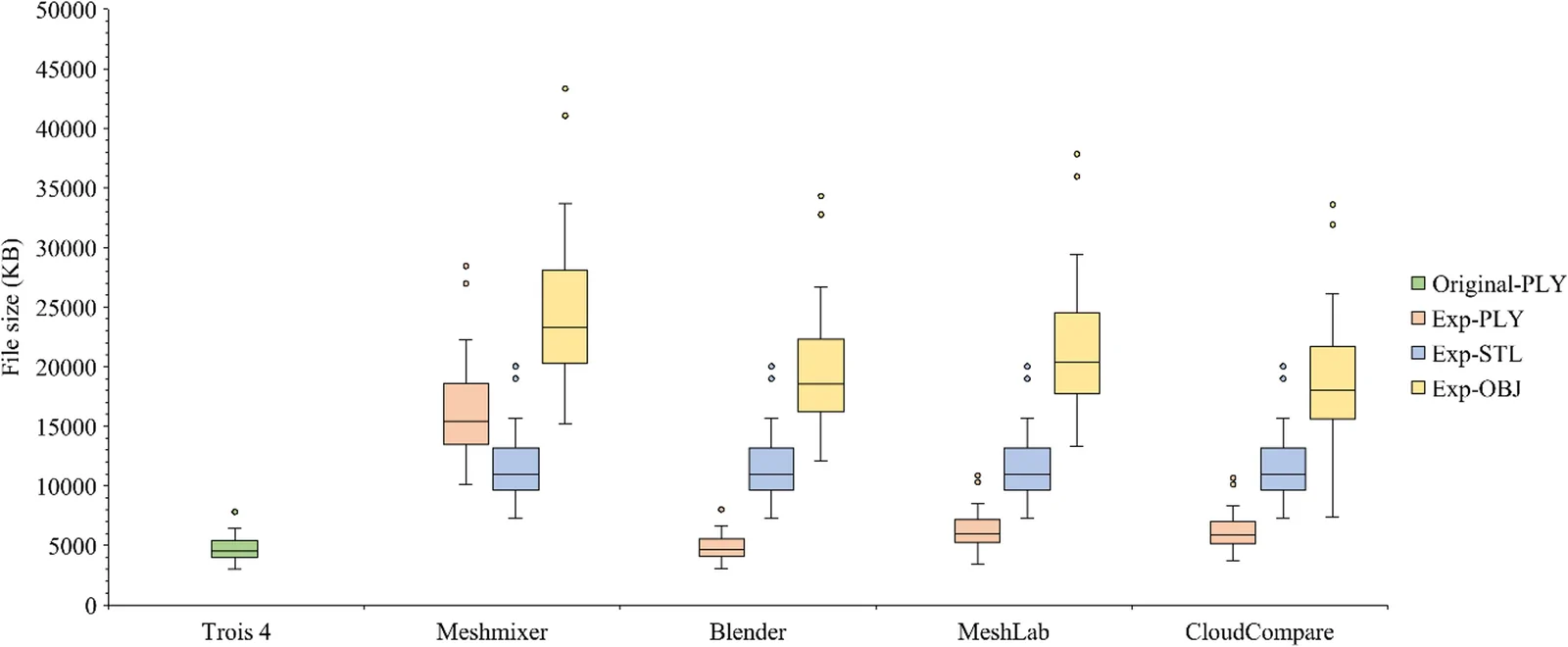
Boxplots of file sizes (KB) for original PLY (Trios 4) and exported PLY, STL, and OBJ files using different software. Boxes show interquartile ranges with medians; whiskers indicate range and dots represent outliers. OBJ files demonstrated the largest file sizes across platforms, followed by STL and PLY formats

The loading and export times of intraoral scan meshes were differed significantly depending on the file format (*p* < 0.001). Original PLY files were loaded into MeshLab fastest with median and interquartile range (IQR) of 293, (76.25) MS, followed by STL 1086, (377) MS, and OBJ 1603, (483) MS (Fig. 3). File exporting times from the reference PLY into different formats also varied. PLY file format showed the fastest export times across all formats with a median of 627, (153.25) MS, whereas STL and OBJ required longer export durations (950, (279.75) MS and 51639, (19344) MS, respectively). These results indicate that file format influences workflow efficiency, with PLY files generally exhibiting superior parsing performance. In addition, the file size efficiency varied markedly across file formats and exporting software (*p* < 0.001). File sizes were consistently smallest for PLY files for all software except for Meshmixer, followed by STL, while OBJ files exhibited the largest storage footprint for all software due to the inclusion of vertex color and additional metadata (Fig. 4). This variations in file size between exporting software, reflecting differences in internal encoding and export implementation. The median and IQR of the file size and exporting software are included in Table 2.

**Table 2:** Summary of mesh quality metrics for original native PLY files and exported PLY, STL, and OBJ models generated using the evaluated software platforms. Data are presented as median and interquartile range (IQR). Reported variables include file size (KB), number of vertices, number of faces, number of edges, average edge length (mm), and surface area (mm²), along with corresponding *p* values for statistical comparisons

Mesh Files Characteristics		Trois 4	Meshmixer			Blender			MeshLab			CloudCompare			*p*-value
		Original-PLY	Exp-PLY	Exp-STL	Exp-OBJ	Exp-PLY	Exp-STL	Exp-OBJ	Exp-PLY	Exp-STL	Exp-OBJ	Exp-PLY	Exp-STL	Exp-OBJ	
File size (KB) (Median, IQR)		4534.5 (1380.5)	15416.5 (4888.25)	10997.5 (3365)	23,311 (7470)	4646 (1414.25)	10997.5 (3365)	18535.5 (12126)	5985 (1818.5	10997.5 (3365)	20,337 (6545)	5873.5 (1784.5	10997.5 (3365)	17,993 (5839.25)	*p* < 0.001
Number of vertices (Median, IQR)		114289.5 (34570.25)	114,290 (34570.3)	114,290 (34570.3)	114,290 (34570.3)	114,290 (34570.3)	114,290 (34570.3)	114,290 (34570.3)	114,290 (34570.3)	114,290 (34570.3)	114,290 (34570.3)	114,290 (34570.3)	114,290 (34570.3)	114,290 (34570.3)	*p* = 1.000
Number of faces (Median, IQR)		225222.5 (68919.75)	225,223 (68919.8)	225,223 (68919.8)	225,223 (68919.8)	225,223 (68919.8)	225,223 (68919.8)	225,223 (68919.8)	225,223 (68919.8)	225,223 (68919.8)	225,223 (68919.8)	225,223 (68919.8)	225,223 (68919.8)	225,223 (68919.8)	*p* = 1.000
Number of edges (Median, IQR)		339,513 (103450.5)	339,513 (103451)	339,513 (103451)	339,513 (103451)	339,513 (103451)	339,513 (103451)	339,513 (103451)	339,513 (103451)	339,513 (103451)	339,513 (103451)	339,513 (103451)	339,513 (103451)	339,513 (103451)	*p* = 1.000
Edge length average (mm) (Median, IQR)		0.218 (0.01)	0.218 (0.01)	0.218 (0.01)	0.218 (0.01)	0.218 (0.01)	0.218 (0.01)	0.218 (0.01)	0.218 (0.01)	0.218 (0.01)	0.218 (0.01)	0.218 (0.01)	0.218 (0.01)	0.218 (0.01)	*p* = 1.000
Surface area (mm^2^) (Median, IQR)		4302.79 (1093.78)	4302.79 (1093.78)	4302.79 (1093.78)	4302.79 (1093.78)	4302.79 (1093.78)	4302.79 (1093.78)	4302.78 (1093.78)	4302.79 (1093.78)	4302.79 (1093.78)	4302.79 (1093.78)	4302.79 (1093.78)	4302.79 (1093.78)	4302.79 (1093.78)	*p* = 1.000

### Geometric accuracy and scale consistency

Bounding box dimensions along X, Y, and Z axes were highly consistent between the original PLY reference datasets and the derived PLY, STL, and OBJ files. Maximum deviations did not exceed 0.000 mm in any axis, indicating that conversion into different file formats and software tools did not introduce significant scaling distortions (*p* = 1.000). Surface area measurements similarly showed no statistically significant differences between original and exported meshes, demonstrating preservation of global anatomical coverage across all formats and software.

### Mesh resolution and tessellation density

Edge-based metrics revealed that total edge count and average edge length were not statically significant across different file formats and exporting software (*p* = 1.000)) (Table 2). The median and IQR for both total edge count and average edge length for all mesh files were 225223 (68919.8), 0.218 (0.01) mm, respectively, they were consistent with the native scanner resolution. No software introduced significant coarsening or over-refinement of the mesh, indicating that local anatomical detail remained adequately represented.

## Discussion

The present study comparatively evaluated computational performance, geometric accuracy, and mesh resolution of PLY, STL, and OBJ files derived from native intraoral scans and exported using different mesh processing software. The findings demonstrated that file format significantly influenced computational performance parameters, including loading time, export time, and file size, whereas no statistically significant differences were observed in geometric fidelity or mesh resolution across formats and software platforms. Therefore, the null hypothesis was partially rejected: file format affected computational efficiency but did not significantly influence geometric accuracy or tessellation characteristics when default export parameters were applied.

In the present study, the native PLY files exported directly from the Trios 4 intraoral scanner were designated as reference datasets for comparative analysis. It is important to note that these datasets do not represent the true anatomical geometry, as they inherently reflect the limitations of the scanning system, including hardware constraints, sampling resolution, and proprietary surface reconstruction algorithms. Therefore, the native PLY files should be regarded as approximations of the underlying anatomy rather than ground truth models.

With respect to computational performance, native PLY files consistently exhibited superior loading and export efficiency compared with STL and OBJ formats. OBJ files, in particular, demonstrated substantially longer export times and larger file sizes. These findings can be attributed to structural differences between file formats [8]. STL files store only geometric triangulation without color or extended attributes, whereas OBJ and PLY formats may include vertex color and additional metadata. The larger storage footprint and prolonged parsing time observed for OBJ files are consistent with prior reports indicating that text-based file structures and extended attribute storage can increase processing overhead in 3D environments [26]. Although the OBJ format demonstrated comparatively lower computational efficiency and larger file sizes in this study, it retains specific advantages in certain clinical contexts. In particular, its ability to store color and texture information via associated material files may be beneficial for applications where visual detail is important, such as shade matching, soft tissue assessment, and esthetic treatment planning. Therefore, despite its relative inefficiency in data handling, OBJ remains a valuable format in workflows where enhanced visual representation is required. Previous research in digital dentistry has primarily focused on the trueness and precision of intraoral scanners, with comparatively little attention given to format-dependent computational performance following data export. Although investigations in this specific area remain limited, the findings of the present study are consistent with those reported by Doan et al. [9], who evaluated file size, geometric fidelity, and the suitability of STL and PLY formats for long-term clinical storage. Their results demonstrated that PLY format reduced storage requirements while maintaining geometric accuracy, including preservation of color information. Beyond dentistry, studies in the fields of 3D modeling and engineering have similarly shown that file structure, encoding type, and metadata handling can significantly influence software parsing efficiency, memory usage, and processing speed [27]. The present results therefore align with established computational principles while extending their relevance to digital dental workflows, providing clinically applicable evidence regarding the impact of file format on performance without compromising geometric integrity [9].

In contrast, geometric accuracy and scale consistency were preserved across all tested formats and software platforms. Bounding box dimensions and surface area measurements demonstrated complete concordance between reference and derived meshes, with no detectable scaling distortions. Furthermore, edge-based metrics confirmed that mesh resolution and tessellation density remained unchanged following conversion. These results suggest that, when no mesh-altering operations (e.g., decimation, smoothing, or remeshing) are applied, format conversion alone does not compromise geometric fidelity. This observation is consistent with accepted theoretical understanding that format translation without resampling should preserve vertex coordinates and topology. It also supports prior studies indicating that most geometric discrepancies in digital dentistry arise from extensive mesh processes rather than post-export file handling [13, 28, 29].

Average edge length serves as an indirect indicator of local sampling density and, consequently, the ability of a mesh to represent fine anatomical features. In the context of dental applications, adequate sampling is essential for accurately capturing structures such as cusps, fissures, and preparation margins, which are critical for prosthetic fit and occlusal function. Smaller edge lengths correspond to higher mesh resolution and improved representation of these features, whereas larger edge lengths may lead to smoothing or loss of detail. In the present study, the observed average edge lengths remained consistent across file formats and fell within ranges generally considered sufficient for preserving clinically relevant surface detail in prosthetic design and additive manufacturing workflows. The absence of differences in edge count and average edge length further indicates that none of the evaluated software platforms introduced unintended coarsening or over-refinement during export. This is clinically relevant, as preservation of dental anatomical detail is essential for prosthetic design accuracy and additive manufacturing outcomes [30]. These findings suggest that, under the evaluated conditions, file format conversion does not compromise the mesh’s ability to represent essential anatomical structures. The results therefore provide reassurance that commonly used open-source software tools can be safely integrated into digital workflows for format conversion without risking mesh integrity, provided that default settings do not include automated repair or optimization functions.

Technical aspects such as numeric precision and metadata storage differ among file formats and may influence data structure and handling. In this study, these differences did not result in measurable geometric variation under the evaluated conditions. From a clinical perspective, their primary relevance lies in workflow efficiency, visualization capabilities, and compatibility with digital systems rather than direct effects on geometric outcomes. Therefore, while these features remain important from a technical standpoint, their clinical impact should be interpreted in the context of overall workflow performance and integration.

Some discrepancies between computational performance findings and commonly held assumptions in clinical practice may be noted. Although STL is traditionally regarded as the “standard” file for manufacturing, it did not demonstrate superior efficiency compared with PLY in this study. The computational differences can be partially interpreted in light of variations in vertex attribute storage and numeric precision during file export. Although geometric metrics remained statistically unchanged across formats and software, significant differences were detected in loading time, export time, and file size. These findings suggest that disparities arose not from modifications of mesh topology or vertex coordinates, but from differences in how data were encoded. For instance, exporters that utilize 64-bit (double-precision) coordinates instead of 32-bit (single-precision) floats effectively increase the data volume per vertex without improving clinically perceptible accuracy at the scale of intraoral scans. Likewise, automatic inclusion of 32-bit per-vertex normal vectors or additional attributes such as color channels can substantially enlarge file size and increase memory allocation during parsing. This mechanism may explain the consistently larger file sizes and prolonged export durations observed for OBJ files, as well as variations between software platforms despite identical mesh geometry. The superior loading and export performance of native PLY files may therefore reflect more compact attribute storage or binary encoding strategies compatible with the scanner’s original data structure.

From a clinical perspective, the observed differences in computational performance have direct implications for digital workflow efficiency. Faster file loading, processing, and export may facilitate more efficient chairside procedures, reducing delays during intraoral scanning, design, and verification stages. In laboratory settings, smaller file sizes and improved processing speed can help minimize data transfer times and reduce bottlenecks in CAD/CAM workflows. Importantly, the preservation of global geometric properties across file formats ensures that these efficiency gains are not achieved at the expense of clinically relevant morphology. As a result, optimized file handling may contribute to smoother integration between clinical and laboratory stages, potentially shortening turnaround times and enhancing overall patient care.

Several limitations must be acknowledged. First, only one intraoral scanner system was evaluated; therefore, findings may not be directly generalizable to datasets generated by other scanning technologies with different tessellation algorithms, including the evaluated platform (Trios 4 intraoral scanner). Second, only default export parameters were assessed, and the influence of optional mesh repair, compression, or binary versus ASCII encoding was not investigated. Third, computational performance was measured within a single analysis environment, and results may vary depending on hardware configuration or software version. Fourth, the study focused exclusively on surface-based metrics and did not evaluate downstream clinical outcomes such as restoration fit or printing accuracy.

Finally, geometric accuracy and mesh resolution were evaluated using global descriptors, including bounding box dimensions, surface area, total edge count, and average edge length. While these metrics provide useful insights into overall geometric consistency and sampling density, they remain indirect and relatively insensitive to localized shape variations. In particular, subtle morphological changes such as the smoothing of sharp edges, rounding of preparation margins, or minor alterations in fine anatomical features may not be fully captured by these global measures. Furthermore, localized geometric assessment methods, such as point-to-point surface deviation analysis or distance-based heat maps, were not implemented in this study. Therefore, caution is warranted when interpreting the findings in terms of the preservation of local anatomical morphology.

Future research should address these limitations by investigating the influence of binary versus ASCII encoding, compression algorithms, and automated mesh optimization procedures (e.g., repair, smoothing, and remeshing) on both computational and geometric performance. Comparative analyses involving multiple intraoral scanner systems and diverse CAD/CAM platforms would further enhance generalizability. In addition, studies incorporating more aggressive mesh reduction settings and meshes derived from cone-beam computed tomography (CBCT) datasets would provide broader clinical relevance. Evaluating the impact of file format on additive manufacturing accuracy and long-term prosthetic outcomes would also yield clinically meaningful insight. Furthermore, future investigations employing high-resolution, spatially resolved comparison techniques, such as point-to-point deviation analysis and distance-based heat mapping, are warranted to better characterize the effect of file format conversion on fine-scale geometric detail. Finally, future research should also consider the implications of file format characteristics in emerging digital ecosystems, including cloud-based dental platforms and AI-driven prosthetic design workflows. In such environments, factors such as file size, encoding efficiency, and data interoperability may play an increasingly important role in enabling rapid data transfer, real-time collaboration, and automated design processes. Evaluating how different file formats perform within these integrated systems may provide valuable insights into optimizing digital workflows in next-generation dental care.

## Conclusion

Within the limitations of this study, file format significantly affected computational performance but did not influence geometric accuracy or mesh resolution when default export parameters were used. Native PLY files demonstrated superior loading and export efficiency and generally smaller file sizes compared with STL and OBJ formats, supporting their advantage in workflow speed and storage efficiency. However, conversion of intraoral scan datasets into PLY, STL, or OBJ formats using different mesh processing software did not introduce scaling distortions, alter surface area measurements, or modify tessellation density. Bounding box dimensions, total edge count, and average edge length remained consistent across all formats and software platforms, indicating preservation of global geometric properties. These findings should be interpreted within the specific study conditions, including the use of a single intraoral scanner system (Trios 4 intraoral scanner), a limited number of open-source software tools, and default export parameters, with the explicit exclusion of mesh-altering procedures such as repair, decimation, smoothing, or remeshing. Furthermore, as the evaluation metrics employed were primarily global descriptors, caution is warranted when extending these results to the preservation of fine local anatomical details. Therefore, while PLY may provide practical computational benefits, PLY, STL, and OBJ formats can be considered geometrically comparable in terms of global geometric characteristics under controlled conditions. Variations in scanner systems, proprietary CAD/CAM environments, or non-default export settings may yield different outcomes and should be considered in future investigations.

## Data Availability

The data that support the findings of this study are available upon reasonable request from the corresponding author. The data are not publicly available due to privacy or ethical restrictions.
